# Supplementary material for: Excipient Impact on Fenofibrate Equilibrium Solubility in Fasted and Fed Simulated Intestinal Fluids Assessed Using a Design of Experiment Protocol
Source: Pharmaceutics. 2023 Oct 17;15(10):2484. doi: 10.3390/pharmaceutics15102484 (PMC10610309; doi:10.3390/pharmaceutics15102484)
Supplement: Supplementary file 1 [file pharmaceutics-15-02484-s001.zip › pharmaceutics-2532194-supplementary.pdf]

Article

Supplementary Material

The excipients' impact on fenofibrate equilibrium solubility in fasted and fed simulated intestinal fluids assessed using a design of experiment protocol

Supplementary Figure S1. Chemical Structures.

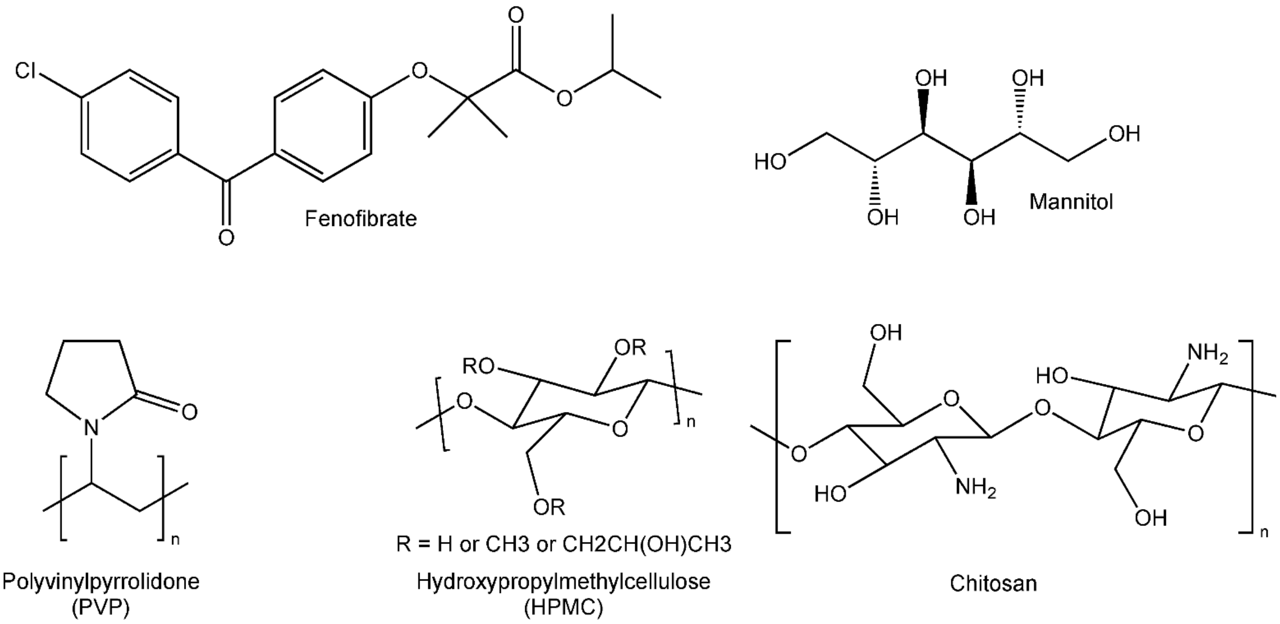

Supplementary Table S1. Stock mixture concentrations (15x lower, mid and upper limits).

Component	Fasted State			Fed State		
	Lower	Middle	Upper	Lower	Middle	Upper
Bile salt	22.5 mM	55.5 mM	88.5 mM	54.0 mM	140 mM	225 mM
Lecithin	3 mM	7.12 mM	11.2 mM	7.50 mM	31.8 mM	56.2 mM
Monoglyceride	1.50 mM	21.8 mM	42.0 mM	15.0 mM	75.0 mM	135 mM
Cholesterol	1.50 mM	2.70 mM	3.90 mM	1.95 mM	8.48 mM	15.0 mM

Supplementary Table S2. Fatty acid volumes (5x upper limit).

Component	Fasted State			Fed State		
	Lower	Middle	Upper	Lower	Middle	Upper
Sodium Oleate	16 µL	248 µL	480 µL	25.6 µL	412.8 µL	800 µL

Supplementary Table S3. Excipients and Concentrations added to DoE Systems.

	Excipient Concentration (mM)			
	Fasted		Fed	
Excipient	Low	High	Low	High
Mannitol	7.32	73.3	9.67	96.7
PVP K12	7.36	73.7	9.73	97.3
PVP K29/32	7.35	73.5	9.71	97.1
HPMC E3	7.35	73.5	9.71	97.1
HPMC E50	6.27	62.6	8.50	85.0
Chitosan	6.64	66.3	9.00	90.0
